# Dynamic changes of the contents of photoprotective substances and photosynthetic maturation during leaf development of evergreen tree species in subtropical forests

**DOI:** 10.1093/treephys/tpad026

**Published:** 2023-03-02

**Authors:** Zheng-Chao Yu, Wei Lin, Wei He, Guan-Zhao Yan, Xiao-Ting Zheng, Yan-Na Luo, Hui Zhu, Chang-Lian Peng

**Affiliations:** Guangdong Provincial Key Laboratory of Biotechnology for Plant Development, Guangzhou Key Laboratory of Subtropical Biodiversity and Biomonitoring, School of Life Sciences, South China Normal University, Guangzhou 510631, China; School of Life Sciences and Food Engineering, Hanshan Normal University, Chaozhou 521041, China; Guangdong Provincial Key Laboratory of Biotechnology for Plant Development, Guangzhou Key Laboratory of Subtropical Biodiversity and Biomonitoring, School of Life Sciences, South China Normal University, Guangzhou 510631, China; Guangdong Provincial Key Laboratory of Biotechnology for Plant Development, Guangzhou Key Laboratory of Subtropical Biodiversity and Biomonitoring, School of Life Sciences, South China Normal University, Guangzhou 510631, China; Guangdong Provincial Key Laboratory of Biotechnology for Plant Development, Guangzhou Key Laboratory of Subtropical Biodiversity and Biomonitoring, School of Life Sciences, South China Normal University, Guangzhou 510631, China; School of Life Sciences and Food Engineering, Hanshan Normal University, Chaozhou 521041, China; Guangdong Provincial Key Laboratory of Biotechnology for Plant Development, Guangzhou Key Laboratory of Subtropical Biodiversity and Biomonitoring, School of Life Sciences, South China Normal University, Guangzhou 510631, China; School of Life Sciences and Food Engineering, Hanshan Normal University, Chaozhou 521041, China; Guangdong Provincial Key Laboratory of Biotechnology for Plant Development, Guangzhou Key Laboratory of Subtropical Biodiversity and Biomonitoring, School of Life Sciences, South China Normal University, Guangzhou 510631, China

**Keywords:** chlorophyll, spatio-temporal replacement

## Abstract

Many studies have investigated the photoprotective and photosynthetic capacity of plant leaves, but few have simultaneously evaluated the dynamic changes of photoprotective capacity and photosynthetic maturation of leaves at different developmental stages. As a result, the process between the decline of photoprotective substances and the onset of photosynthetic maturation during plant leaf development are still poorly understood, and the relationship between them has not been quantitatively described. In this study, the contents of photoprotective substances, photosynthetic pigment content and photosynthetic capacity of leaves at different developmental stages from young leaves to mature leaves were determined by spatio-temporal replacement in eight dominant tree species in subtropical evergreen broadleaved forests. The correlation analysis found that the data sets of anthocyanins, flavonoids, total phenolics and total antioxidant capacity were mainly distributed on one side of the symmetry axis (*y* = *x*), while the data sets of flavonoids, total phenolics and total antioxidant capacity were mainly distributed on both sides of the symmetry axis (*y* = *x*). In addition, the content of photoprotective substances in plant leaves was significantly negatively correlated with photosynthetic pigment content and photosynthetic capacity but was significantly positively correlated with dark respiration rate (Rd). When chlorophyll accumulated to ~50% of the final value, the photoprotective substance content and Rd of plant leaves reached the lowest level, and anthocyanins disappeared completely; in contrast, the photosynthetic capacity reached the highest level. Our results suggest that anthocyanins mainly play a light-shielding role in the young leaves of most plants in subtropical forests. In addition, 50% chlorophyll accumulation in most plant leaves was the basis for judging leaf photosynthetic maturity. We also believe that 50% chlorophyll accumulation is a critical period in the transition of plant leaves from high photoprotective capacity (high metabolic capacity, low photosynthetic capacity) to low photoprotective capacity (low metabolic capacity, high photosynthetic capacity).

## Introduction

It has been found that in approximately one-third of plant species of tropical forests (Lee and Collins 2001, Dominy et al. 2002) and one-third of woody plant dominant tree species of subtropical forests (Zhang et al. 2019, Gong et al. 2020), young leaves will turn red because of the accumulation of anthocyanins. The investigation of perennial woody plants by Price and Sturgess (1938) and of herbaceous plants in temperate areas by Chalker-Scott (1999) also found that the young leaves turn red, and the red gradually disappears with leaf maturity. Therefore, what is the physiological significance of the accumulation of anthocyanins in the young leaves of these plants? At present, there is no unified consensus in academia. In recent years, the photoprotection hypothesis of anthocyanins in leaves has been supported by many scholars (Feild et al. 2001, Cai et al. 2005, Hughes and Smith 2007, Li et al. 2008, Logan et al. 2015, Zhang et al. 2016, Buapet et al. 2017, Tattini et al. 2017, Zheng et al. 2021). This is because anthocyanins not only achieve optical masking of chloroplasts to reduce oxidative stress by reducing the absorption of light energy (Feild et al. 2001, Steyn et al. 2002, Landi et al. 2014, Nichelmann and Bilger 2017, Zheng et al. 2021), but also reduce oxidative damage to leaves by scavenging reactive oxygen species (ROS) (Neill et al. 2002*a*, 2002*b*, Neill and Gould 2003). Although anthocyanins can act as antioxidants or light-shielding agents to play a photoprotective role during leaf development, the accumulation of anthocyanins alone cannot meet the needs of young leaves for photoprotection; therefore, diversified means are needed to adjust and achieve the optimal effect of photoprotection. Some small-molecule antioxidants, such as total phenolics, flavonoids, ascorbic acid and glutathione (Karageorgou and Manetas 2006, Salgado et al. 2008, Ghasemzadeh et al. 2010, Mustafa et al. 2010, Tattini et al. 2010, Huang et al. 2016, Zhang et al. 2016, 2018*a*, 2018*b*, Henry-Kirk et al. 2018), and some antioxidant enzymes, such as superoxide dismutase (SOD), catalase (CAT), glutathione reductase (GR) and peroxidase (SOD) (Silva et al. 2010, 2013, Favaretto et al. 2011, Yu et al. 2021*b*, Lin et al. 2022), etc., also play an important role in contributing to the photoprotection requirement. However, although it has been demonstrated that these photoprotective substance play an important photoprotective role during leaf development, few studies have explored the dynamic changes of photoprotective substances content and photosynthetic maturity in leaves at different developmental stages.

What are the physiological changes in plant leaves during the maturation process? First, the photoprotective ability of the leaves will decrease. Studies have confirmed that over leaf development, the photoprotective potential of plant leaves is inversely proportional to photosynthetic capacity (Zhang et al. 2018*a*). The comprehensive photoprotective potential of young leaves is high, largely relying on the synthesis of a large number of photoprotective substances (Steyn et al. 2002, Karageorgou and Manetas 2006, Tattini et al. 2010, Landi et al. 2014, Xu et al. 2017). For example, Zhu et al. (2018) found that the young leaves of *Acmena acuminatissima* achieve photoprotection through non-photochemical quenching (NPQ) in summer and accumulation of anthocyanins in winter. Zhang et al. (2018*a*, 2018*b*) found that the photoprotective potential of young leaves in a high-light environment mainly depends on the synthesis of flavonoids and total phenolics. Second, the photosynthetic pigment content and photosynthetic capacity of the leaves gradually increase as young leaves mature (Wullschleger and Oosterhuis 1990). It has been suggested that a marker of young leaf maturity is related to the dry matter accumulation per unit leaf area and to the thickness of mesophyll cells and cell walls in the leaf (Miyazawa et al. 1998, Wright et al. 2004). As leaves mature, dry matter content per unit leaf area increases significantly (Mooney and Gulmon 1992), as well as the size and number of mesophyll cells and the leaf thickness (Nobel et al. 1975, Flexas and Medrano 2002). The increase in leaf thickness is accompanied by the elongation of palisade and spongy parenchyma, which can increase the area of exposure of cells to intercellular air spaces to facilitate photosynthesis of plants (Zhai et al. 2020). On the other hand, it is beneficial to the uniform distribution of light energy in the leaves and improves the photosynthetic efficiency of plants (Vogelmann and Martin 1993, Dos Santos et al. 2021). In addition, as the leaves mature, the surface area of the cell wall and the thickness of the mesophyll cells increase, which increases the contact area with the chloroplasts, creating a larger space for chloroplast accumulation (Oguchi et al. 2003). This reduces the resistance of CO_2_ diffusion in the cytoplasm and increases the utilization rate of CO_2_ in plants (Niinemets et al. 2005, Terashima et al. 2011, Tholen et al. 2012). Marchi et al. (2008) also believed that the difference in photosynthetic potential from young leaves to mature leaves is related to the formation of mesophyll cells because the increase of mesophyll cells is often accompanied by a large increase in chlorophyll and Rubisco content. Finally, as young leaves mature, their energy demand gradually decreases. In the young leaf stage, the leaf is undergoing the construction process. To satisfy this need, the leaf increases the energy demand and therefore has a higher dark respiration rate (Rd) (Lambers et al. 1983). After the leaf matures, the required construction demand decreases, and Rd decreases (Marchi et al. 2008). Miyazawa et al. (2003) also believed that photosynthetic maturation mainly depends on the process construction of the photosynthetic apparatus, and that the construction of the cell wall also occurs when the photosynthetic capacity of leaves increases (Jurik, 1986). Therefore, leaves consume the most energy in the young leaf stage, and energy demand decreases gradually with increasing maturity. Although the above studies have described the physiological changes from young leaves to mature leaves, there is rarely an accurate quantitative standard to quantitatively describe the relationship between these physiological changes and photosynthetic maturity, including the changes of photoprotective substances, photosynthetic pigments and photosynthetic capacity, prompting the present investigation.

**Table 1:** Information on the eight selected tree species of subtropical evergreen broadleaf forests and the phenotypes of young leaves during the experiment.

Species	Family	Growth form	Young leaf phenotype
*Schima superba* Gardn. et Champ.	Theaceae	Tree	Light green
*Castanopsis chinensis* Hance	Fagaceae	Tree	Light green
*Castanopsis fissa* (C. ex B.) R. et W.	Fagaceae	Tree	Slightly red
*Machilus chinensis* (Champ. ex Benth.) Hemsl.	Lauraceae	Tree	Red
*Cryptocarya chinensis* (Hance) Hemsl.	Lauraceae	Tree	Red
*Cryptocarya concinna* Hance	Lauraceae	Tree	Red
*Syzygium acuminatissimum* (Blume) DC.	Myrtaceae	Tree	Red
*Syzygium rehderianum* Merr. et Perry	Myrtaceae	Tree	Red

## Materials and methods

### Study site and plant material

This study was conducted at the Biological Park Base of South China Normal University (113°20′59.05′′ E, 23°8′22.39′′ N) in Guangzhou, Guangdong Province, China. In this study, eight dominant tree species in subtropical evergreen broadleaf forest were selected as materials, including *Castanopsis fissa*, *Castanopsis chinensis*, *Schima superba*, *Machilus chinensis*, *Cryptocarya chinensis*, *Cryptocarya concinna*, *Syzygium acuminatissimum* and *Syzygium rehderianum* (see Table 1 for tree species information). The seedlings were collected from Dinghushan National Nature Reserve (112°30′39″–112°33′41″ E; 23°09′21″–23°11′30″ N), Zhaoqing City, Guangdong Province. In 2013, 20 saplings of each of the 8 tree species were planted in a potted manner in the Biological Garden Base of South China Normal University. The planting medium used was clay loam and peat soil (volume ratio of 3:1). The plastic basin of potted plants is 50 cm in diameter and 35 cm in height. Both the climate of the planting site and the original climate of the plants belong to the subtropical humid monsoon climate, with an annual average temperature of 22.8 °C and an average annual rainfall of 1736 mm. The plants were watered regularly to keep the soil moist and supplied with ammonium sulfate (Changzhou Shanglian Chemical Co., Ltd; CAS: 7783-20-2 Changzhou City, Jiangsu Province, China) fertilizer every 3 months at a dose of 20 g per pot. The height of these tree seedlings was ~1 m when they were transplanted from Dinghu Mountain, and the experiment was conducted from October 2020 to March 2021 after 7 years of planting. During the experiment, the average temperature was 16.2 °C, and the maximum light intensity during the day was 1800 μmol m^−2^ s^−1^. The selected study objects were leaves at different developmental stages, from just growing out (~2 days) to full maturity: 150–200 leaves at different developmental stages of each species were randomly selected for the determination of physiological and biochemical indices. The anthocyanin content, flavonoid content, total phenolic content, total antioxidant capacity (TAC), photosynthetic pigment content and photosynthetic capacity must be determined on each leaf to ensure that all indices on each leaf can be matched. After we measure the gas exchange parameters of the leaves, we picked the leaves from the tree, and then a 6 mm diameter punch to obtain six leaf disks. Each pair of leaf disks was wrapped with aluminum foil, marked and stored in a liquid nitrogen tank, for later measurement of other indicators.

### Determination of gas exchange parameters

Gas exchange parameters of leaves at different developmental stages were determined using a portable infrared gas analyzer Li-6400 (Li-COR, Inc., Lincoln, NE, USA) on consecutive sunny mornings (8:30–11:30 a.m.). The instrument was tested before measurement to ensure the normal operation of the instrument, and then the measurement of the subsequent gas exchange parameters was carried out. During measurement, the surrounding average temperature was ~22 °C, the relative air humidity was 48% and the average air CO_2_ concentration was 400 μmol mol^−1^. Additionally, during measurement, the CO_2_ concentration in the leaf chamber was consistent with the CO_2_ concentration in the surrounding environment. The light source for the leaf chamber was LED light (red and blue light in a ratio of 9:1). Each leaf was induced with a saturated light intensity of 800 μmol m^−2^ s^−1^ for 5–8 min (during which time the plants could reach the maximum net photosynthetic rate at this light intensity and without photoinhibition). When the photosynthetic parameters displayed on the instrument were stable, the net photosynthetic rate (Pn) of the leaves was recorded. The measurement steps of Rd were the same as measurement steps of Pn, after setting the light intensity to 0 μmol m^−2^ s^−1^.

### Determination of pigment content

The determination of anthocyanin content was based on the method of Yu et al. (2021*a*) with a slight modification. The leaves used for the determination of anthocyanin content were the same leaves used to measure the gas exchange parameters. Anthocyanins from two leaf disks (6 mm in diameter) were extracted and placed in a 2 ml centrifuge tube with 0.8 ml of 1% HCl-methanol (v/v). The extraction conditions were 4 °C for 24 h. To remove chlorophyll and extract anthocyanins, equal volumes of chloroform (trichloromethane) and 1/2 volume of distilled water were added to the extract. After full mixing, the solution appeared layered (anthocyanins were dissolved in the upper methanol–water phase, and chlorophyll was dissolved in the lower chloroform phase). The upper layer solution was pipetted into a cuvette, to measure the absorbance at 530 nm using a UV2450 spectrophotometer (Shimadzu, Tokyo, Japan). Different concentration gradients of cyanidin-3-O-glucoside were used to establish a standard curve to calculate the content of anthocyanins.

The extraction of photosynthetic pigments was carried out according to the method of Zhang et al. (2016). From the same leaf used for measuring gas exchange parameters and anthocyanin content, two leaf disks were obtained with a 6 mm diameter hole punch. The leaf disks were placed into a 4 ml centrifuge tube and a small amount of liquid nitrogen was added to smash leaf disks into powder. Then 2 ml of 80% acetone was added into the centrifuge tube for extraction in the dark for 24 h (shaken every 8 h). After the chlorophyll in the leaves was completely dissolved, the supernatant was pipetted into a cuvette, and the absorbance values were recorded at 663, 645 and 470 nm with a UV2450 spectrophotometer (Shimadzu, Tokyo, Japan). The photosynthetic pigment content was calculated according to the formula of Wellburn (1994).

### Determination of flavonoids, total phenolics and TAC

Total phenolics and total small-molecule antioxidants in plant leaves were all extracted with 95% methanol solution. From the same leaf used for measuring gas exchange parameters, anthocyanin content and photosynthetic pigment content, two leaf circles were obtained with a 6 mm diameter hole punch. The leaf disks were placed into a centrifuge tube pre-filled with 2 ml of 95% methanol solution. The flavonoids, total phenolics and TAC were measured after soaking in a 4 °C refrigerator for 48 h. The determination of flavonoid content was optimized according to the method of Heimler et al. (2005). Total phenolic content was determined in a similar way to that of Ainsworth and Gillespie (2007). Total antioxidant capacity (TAC) was determined according to the method of Zhang et al. (2018*a*).

### Data processing and analysis

The distributions of all variables were checked. To ensure the homogeneity of the data of each indicator of each species, before performing regression analysis, each indicator data conduct standardized deal with, that is, normalized deal with (relative content treatment: each index in each species was treated with a maximum value of 100%; since the Pn of the young leaves had a negative value, the smallest negative value was set to −100%, and the largest value was set to +100% for processing), so as to eliminate the data differences caused by the differences between different tree species and ensure that the data could be compared to the same extent. The preliminary data processing was completed in Excel 2016, and then statistic tests were performed in SPSS 18 (SPSS Inc., Chicago, IL, USA) software. Sigmaplot 14 (SYSTAT software, San Jose, CA, USA) software was used to perform the linear regression analysis and inverse first order regression analysis of bivariate relationships and for plotting. In addition, structural equation modeling (SEM) (Grace, 2006) was used to quantify the correlation between photoprotective substances, photosynthetic pigments and photosynthetic capacity. The structural equation model was analyzed using AMOS 22.0 (AMOS Development Corporation, Spring House, PA, USA). When the Chi-square test of the model satisfied *P* > 0.05 and RMSEA ≤  0.05, the model was considered to pass the test, and the model results were credible.

## Results

### Relationship among anthocyanins, flavonoids, total phenolics and TAC

**Figure 1:**
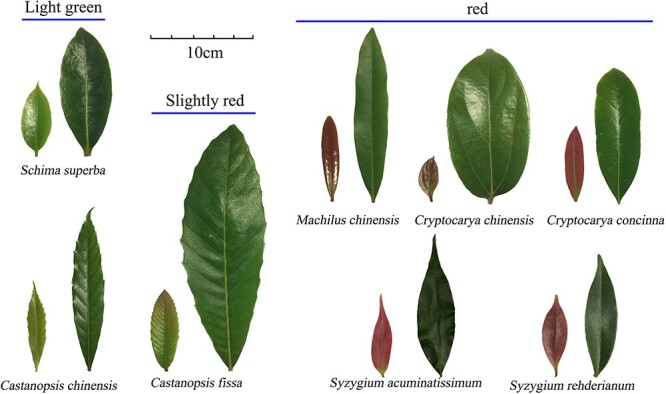
Appearances of young and mature leaves of eight subtropical broadleaf evergreen forest species during the experiment.

**Figure 2:**
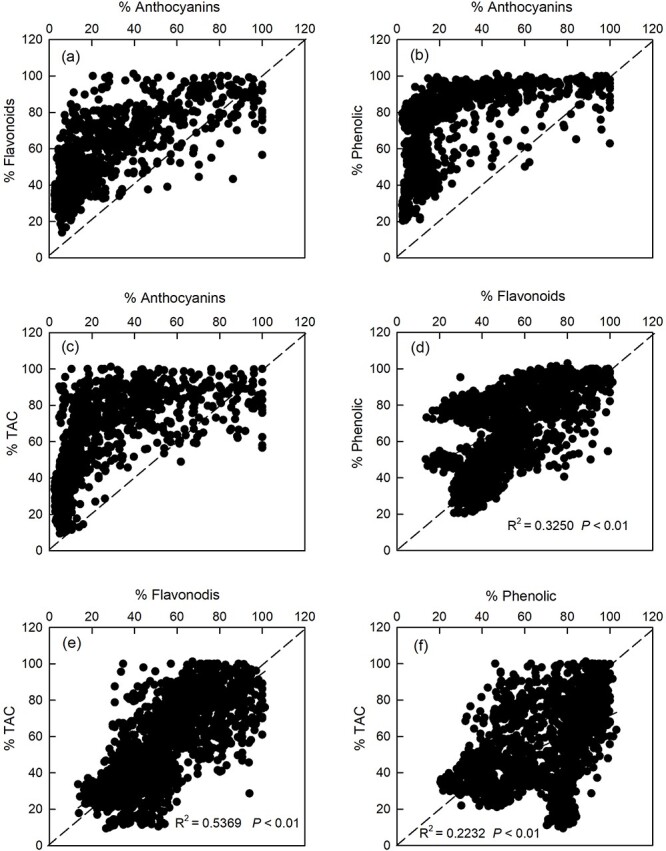
Relationships between anthocyanin content, flavonoid content, total phenolic content and total antioxidant capacity (TAC) in leaves of eight tree species at different developmental stages in subtropical evergreen broadleaf forest. (a) Relationship between anthocyanins and flavonoids; (b) relationship between anthocyanins and total phenolic; (c) relationship between anthocyanins and TAC; (d) relationship between flavonoids and total phenolic; (e) relationship between flavonoids and TAC; and (f) relationship between total phenolic and TAC. The relative content (%) on the *x*-axis and *y*-axis was the content of each index of each species relative to the maximum value of this index for this species.

In the early stage of leaf development, the young leaves of most plants turn red due to the accumulation of anthocyanins (Figure 1). This is because anthocyanins, flavonoids and total phenolics are all effective photoprotective substances. However, we observed that the correlations between anthocyanins and other photoprotective substances (flavonoids, total phenolics and TAC) were different from the correlations between flavonoids, total phenolics and TAC (Figure 2). The correlation data sets of anthocyanins and flavonoids (Figure 2a), total phenolics (Figure 2b) and TAC (Figure 2c) were almost fully distributed on one side of the symmetry axis (*y* = *x*). In contrast, the correlation data sets of flavonoids, total phenolics and TAC (Figure 2d and e) were all distributed on both sides of the symmetry axis (*y* = *x*) and showed a significant positive correlation (*P* < 0.05).

### Relationship between photosynthetic pigments and photoprotective substances

**Figure 3:**
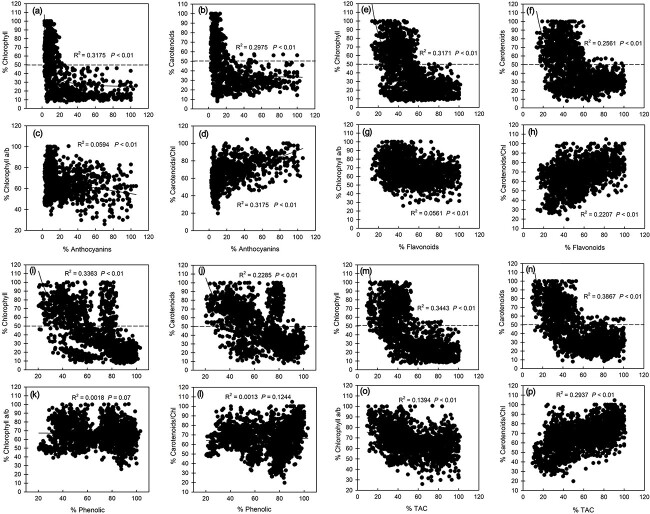
Relationship between photosynthetic pigments (total chlorophyll content, carotenoid content, chlorophyll a/b and Car/Chl) and anthocyanin content, flavonoid content, total phenolic content and TAC in the leaves of eight tree species at different developmental stages in subtropical evergreen broadleaf forest. The relative content (%) on the *x*-axis and *y*-axis was the content of each index of each species relative to the maximum value of this index for this species.

With the increasing maturity of leaves and the accumulation of photosynthetic pigments, the anthocyanins in leaves gradually disappeared. Our results show that %Anthocyanins did not show a simple linear correlation with %Chlorophyll and %Carotenoids but rather an inverse first order relationship (*P* < 0.05) (Figure 3a and b). After further analysis, it was found that the anthocyanins almost completely disappeared when %Chlorophyll and %Carotenoids reached ~50% of the final value (Figure 3a and b). In addition, %Anthocyanins showed a significant negative correlation with %Chlorophyll a/b (%Chl a/b) (*P* < 0.05) (Figure 3c), but a significant positive correlation with % Carotenoids/Chlorophyll (%Car/Chl) (*P* < 0.05) (Figure 3d). Similarly, %Flavonoids, %Phenolics and %TAC all had inverse first order relationships with %Chlorophyll and %Carotenoids (*P* < 0.05) (Figure 3e, f, i, j, m and n). In addition, %Flavonoids, %Phenolics and %TAC were significantly negatively correlated with %Chl a/b (*P* < 0.05) (Figure 3g, k and o). In contrast, %Flavonoids and %TAC showed a significant positive correlation (*P* < 0.05) with %Car/Chl (Figure 3h and p), while %Phenolics had no significant relationship with %Car/Chl (Figure 3l). Likewise, %Flavonoids, %Phenolics and %TAC all decreased to a minimum when %Chlorophyll and %Carotenoids accumulated to ~50% (Figure 3e, f, i, j, m and n).

### Relationship between photoprotective substances, photosynthetic pigments and photosynthetic capacity

**Figure 4:**
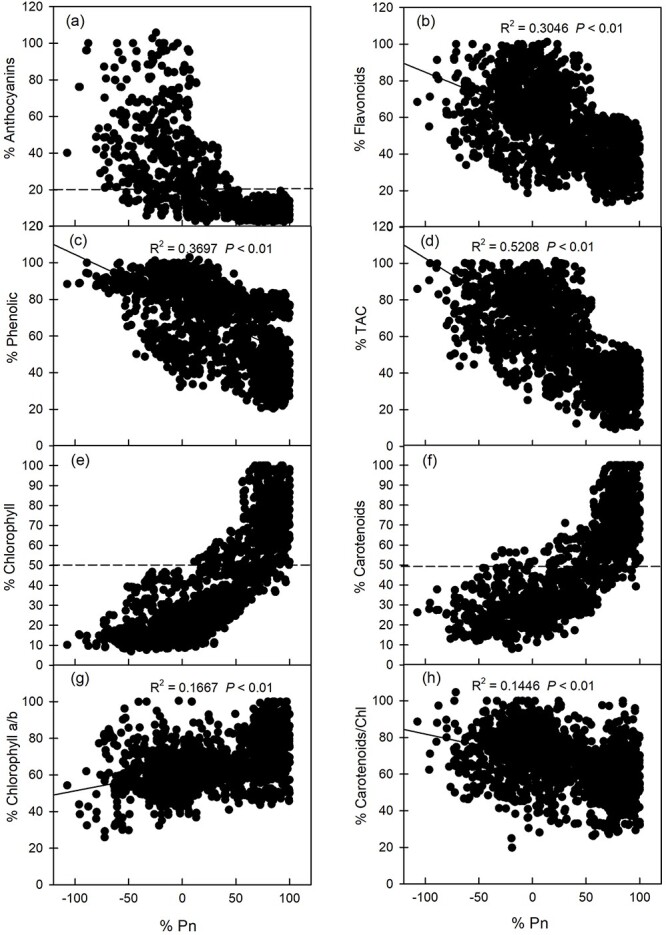
Relationship between photosynthetic capacity with anthocyanin content, flavonoid content, total phenolic content, TAC, total chlorophyll content, carotenoid content, Chl a/b and Car/Chl in the leaves of eight tree species at different developmental stages in subtropical evergreen broadleaf forest. The relative content (%) on the *x*-axis and *y*-axis was the content of each index of each species relative to the maximum value of this index for this species.

The plants did not perform efficient photosynthesis (organic matter accumulation) at the young leaf stage because their %Pn was negative. As the leaves matured, anthocyanins gradually disappeared, and the photosynthetic capacity gradually increased and reached a maximum when the anthocyanins disappeared completely (Figure 4a). In addition, %Flavonoids, %Phenolics and %TAC were significantly negatively correlated with %Pn (*P* < 0.05) (Figure 4b–d). From the results of the relationship between photosynthetic pigments and photosynthetic capacity, it was found that the %Pn of plant leaves could reach the maximum when %Chlorophyll accumulated to ~50% (Figure 4e). The results for %Carotenoids were consistent with those for %Chlorophyll (Figure 4f). As leaves matured, %Pn had a significant positive correlation (*P* < 0.05) with %Chl a/b (Figure 4g), but a significant negative correlation (*P* < 0.05) with %Car/Chl (Figure 4h).

### Relationship between photoprotective substances, photosynthetic pigments and Rd

**Figure 5:**
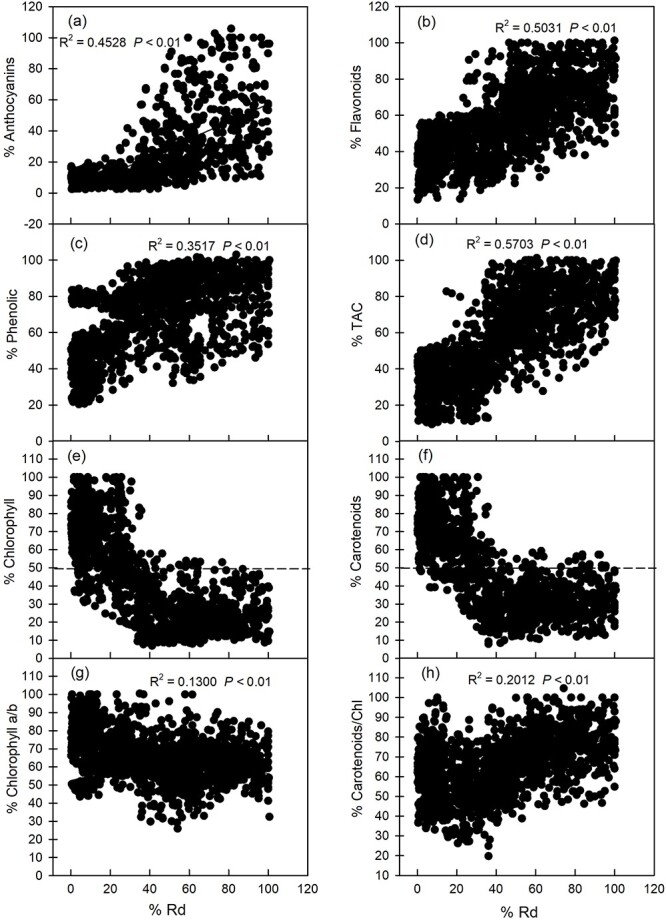
Relationship between dark respiration rate with anthocyanin content, flavonoid content, total phenolic content, TAC, total chlorophyll content, carotenoid content, Chl a/b and Car/Chl in the leaves of eight tree species at different developmental stages in subtropical evergreen broadleaf forest. The relative content (%) on the *x*-axis and y-axis was the content of each index of each species relative to the maximum value of this index for this species.

In addition, %Anthocyanins, %Flavonoids, %Phenolics and %TAC all had significant positive correlations with %Rd (*P* < 0.05) (Figure 5 a–d). Figure 5 shows that the increase in photoprotective material content is accompanied by a significant increase in Rd. As the leaves matured, the increase of %Chlorophyll and %Carotenoids, the %Rd of plants gradually decreased. The %Rd of plants reached a minimum when the %Chlorophyll and %Carotenoids increased above ~50%. This can be observed from the correlation between %Rd with %Chlorophyll and %Carotenoids (Figure 5e and f). %Rd had a significant negative correlation (*P* < 0.05) with %Chl a/b (Figure 5g), but a significant positive correlation (*P* < 0.05) with %Car/Chl (Figure 5h), which was contrary to the correlation between %Pn with %Chl a/b and %Car/Chl.

### Photoprotective substances, photosynthetic pigments and photosynthetic capacity correlation networks

**Figure 6:**
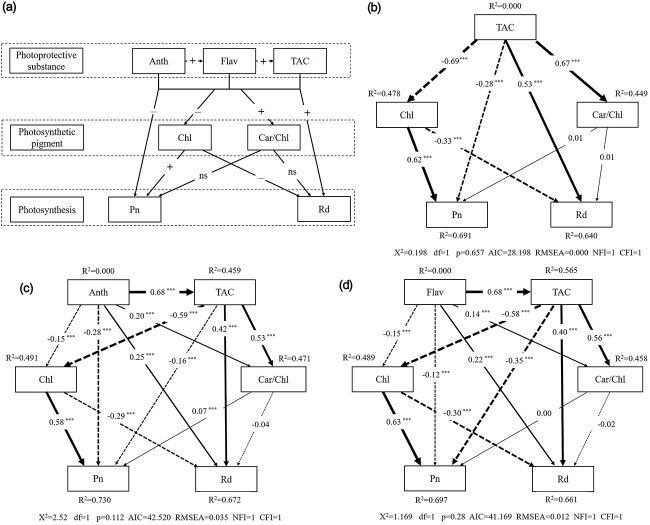
Path model of the relationship between various physiological indexes of leaves of eight tree species at different developmental stages in subtropical evergreen broadleaf forest. (a) General conceptual framework for the relationship between various physiological indicators. Physiological indicators were divided into three groups: photoprotective substances, photosynthetic pigments and photosynthetic capacity. (b) Exploring the relationship model of TAC with total chlorophyll content, Car/Chl, photosynthetic rate (Pn) and dark respiration rate (Rd). (c) Exploring the relationship model of anthocyanin content with TAC, total chlorophyll content, Car/Chl, Pn and Rd. (d) Relationship model of flavonoid content and TAC, total chlorophyll content, Car/Chl, Pn and Rd. Each model tests a subset of the conceptual framework. The numbers on the arrows are the standardized path coefficients. The R^2^ value next to each variable represented the proportion of change explained. Anth = anthocyanin; Flav = flavonoid; TAC = total antioxidant capacity; dashed line represented negative correlation; solid line represented positive correlation; the thickness of the line represents the strength of the correlation.

Based on the conceptual framework model in Figure 6a, we first fitted the relationship of TAC with photosynthetic pigments and photosynthetic capacity using a model function, and it had a fitting index Root Mean Square Error of Approximation (RMSEA) of 0.000 and Akaike information criterion (AIC) of 28.2 (Figure 6b). Total antioxidant capacity (TAC) was directly negatively correlated with Chl (−0.69) and Pn (−0.28) but was directly positively correlated with Car/Chl (0.67) and Rd (0.53); Chl was directly positively correlated with Pn, but had a direct negative correlation with Rd, while Car/Chl had no effect on Pn or Rd. Additionally, the relationship of Anth with TAC, photosynthetic pigment content and photosynthetic capacity was fitted using a model function, and the fitting index RMSEA was 0.035 and AIC was 42.5 (Figure 6c). The results showed that Anth had a direct positive correlation with TAC (0.68), Car/Chl (0.20) and Rd (0.25) but a direct negative correlation with Chl (−0.15) and Pn (−0.28). Finally, the relationship of Flav with TAC, photosynthetic pigments and photosynthetic capacity was carried out, and the fitting index RMSEA was 0.012 and AIC was 41.169 (Figure 6d). The results were similar to those of Anth. Flav had a direct positive correlation with TAC (0.68), Car/Chl (0.14) and Rd (0.22), but a direct negative correlation with Chl (−0.15) and Pn (−0.12).

## Discussion

### Role of anthocyanins in photoprotection

Previous studies have shown that young leaves can benefit from the accumulation of anthocyanins during maturation (Cai et al. 2005, Zhang et al. 2016, 2018*a*, Yu et al. 2021*b*), because young leaves are relatively fragile and more vulnerable to light stress than mature leaves (Choinski et al. 2003, Hughes et al. 2007). Therefore, young leaves achieve photoprotection by accumulating anthocyanins. Thus, what kind of photoprotective role does the reddening of young leaves play: antioxidant or light attenuator? The correlation analysis between different photoprotective substances in young leaves at different developmental stages provided us with evidence. We found that the data sets of anthocyanins and flavonoids, total phenolics and TAC were mainly distributed on one side of the symmetry axis (*y* = *x*) (Figure 2a–c), while the data sets of flavonoids, total phenolics, and TAC were all distributed on both sides of the symmetry axis (*y* = *x*) (Figure 2d–f), and all showed a downward trend with leaf maturity. This shows that anthocyanins, flavonoids and total phenolics were all effective photoprotective substances at the young increasing leaf stage, but it was apparent that anthocyanins had different photoprotective effects from flavonoids and total phenolics. Anthocyanins mainly play a light-shielding role during the development of young leaves rather than an antioxidant role such as flavonoids and total phenolics play. If anthocyanins were to play an antioxidant role only, then the data sets between it and flavonoids, total phenolics and TAC should be distributed on both sides of the axis of symmetry (*y* = *x*) in theory. In fact, this was not the case. In addition, we randomly selected the eight dominant tree species we selected in subtropical evergreen broadleaf forests, so we could indirectly infer the physiological significance of anthocyanin accumulation in young leaves of most woody plants in subtropical forests: it plays a light-attenuating role. Perhaps this conclusion can also be extrapolated to most plants whose young leaves turn red. Of course, this needs to be verified by subsequent data collection. However, our idea of distinguishing the photoprotective effect of anthocyanins may be an effective method.

### Relationship between photoprotective substance content and photosynthetic pigment content in leaves

The maturation of young leaves was accompanied by the accumulation of photosynthetic pigments and the reduction of photoprotective substances. Thus the %Anthocyanins (Figure 3a and b), %Flavonoids (Figure 3e and f), %Phenolics (Figure 3i and j) and %TAC (Figure 3m and n) of young leaves exhibited a significant inverse first order relationship with %Chlorophyll and %Carotenoids during the maturation process. This shows that the decrease of photoprotective substances during the maturation of a young leaf might correspond to an increased demand for photosynthetic pigments, because the accumulation of photosynthetic pigments could efficiently use more light energy, so as to reduce the demand for photoprotective substances. This could also be seen from the significant negative correlation of %Chla/b with %Anthocyanins, %Flavonoids and %TAC (Figure 3c, g and o) and the significant positive correlation with %Pn (Figure 4g). This is because Chla/b embodies the light-harvesting ability of plants. On the contrary, photoinhibition is caused by excessive light intensity entering the photosynthetic mechanism, and the accumulation of photoprotective substances is a means by which to eliminate photoinhibition. In addition to a gradual decrease in anthocyanins, flavonoids, total phenolics and TAC during young leaf development, the Car/Chl also decreased significantly (Figure 4h), and %Car/Chl was significantly positively correlated with %Anthocyanins, %Flavonoids and %TAC (Figure 3d, h and p). Car/Chl also provides a common means of photoprotection, which relies on heat dissipation from the xanthophyll cycle. Under environmental stress, plants can dissipate excess light energy in the photosynthetic system in the form of heat energy through the xanthophyll cycle to achieve photoprotection (Demmig-Adams and Adams 1992, Leipner et al. 1997, Zhu et al. 2018). This suggested that the photoprotection requirements contributed by Car/Chl also decreased as the leaves matured, which was consistent with the results of Hughes et al. (2007). Notably, the reduction of photoprotective substances and the increase of photosynthetic pigments were not all negatively correlated during the maturation process of young leaves, and ~50% chlorophyll accumulation was the clear dividing line. When the accumulation level of chlorophyll was lower than 50%, the content of photoprotective substances decreased with the accumulation of chlorophyll, and the content of photoprotective substances reached the lowest level when the accumulation of chlorophyll reached about 50%. In contrast, the content of photoprotective substances changed little with the accumulation of chlorophyll above 50% (Figure 3a, e, i and m). Similar studies have also been reported by other researchers, who believe that the disappearance of anthocyanins in young leaves corresponds to the accumulation of 50% of photosynthetic pigments (Hughes et al. 2007). However, those results were limited to studies of anthocyanins only; changes in the contents of other photoprotective substances were not measured. In addition, the number of leaves at different developmental stages and of tree species they collected were fewer. Thus, the results of our study may be relatively more systematic and complete.

### Relationship between photoprotective substance content and Rd in leaves

The accumulation of a large amount of photoprotective substances in young leaves incurs costs. The synthesis of these photoprotective substances requires precursor material and energy supply, which has been confirmed by many studies (Vaknin et al. 2005, Kyparissis et al. 2007, Zhang et al. 2016, Yu et al. 2019). In addition, cells in young leaves also carry out other vigorous physiological and metabolic processes to sustain such activities, so they need to consume a large amount of energy for maintenance (Guo et al. 2018). This explains why %Anthocyanins, %Flavonoids, %Phenolics, %TAC and %Car/Chl were significantly positively correlated with %Rd (Figure 5a–d and h; Figure 6b–d). Importantly, Rd decreased gradually with increasing chlorophyll content, but this trend was not uniform. In the early stage of leaf development, Rd gradually decreased with the accumulation of chlorophyll, and Rd decreased to the lowest level when chlorophyll accumulated to 50%. The subsequent increase in chlorophyll did not cause significant changes in Rd (Figure 5e and f). Interestingly, the accumulation of chlorophyll also requires energy consumption in theory. Therefore, one wonders whether reality contradicts theory. In fact, this was not the case. The real reason for this change trend was not the accumulation of chlorophyll but rather the photosynthetic maturity of the leaves. Here, chlorophyll was only used to reflect photosynthetic maturity. After the photosynthetic maturity of plant leaves is reached, the demand for photoprotective substances would be reduced, which could also be observed from the significant negative correlation of the content of photoprotective substances with chlorophyll content and photosynthetic energy. On the other hand, some structures in the corresponding leaf are well developed after leaf photosynthetic maturity, which makes the physiological and metabolic activities of cells in the leaf stable, so the energy consumption is reduced.

### Relationship between photoprotective substance content and photosynthetic capacity of leaves

Aside from an increase in chlorophyll content, an increase in photosynthetic capacity is also a characteristic of leaf maturation during leaf development (Zhang et al. 2018*a*). Photosynthesis is an important physiological process in plant growth (Bloor and Grubb 2003, Kelly et al. 2009). Due to the immaturity of photosynthetic pigments and photosynthetic machinery, it will not perform effective photosynthesis (accumulation of organic matter) in plant leaves in the young leaf stage (Zhang et al. 2016). However, an effective photoprotection strategy will be formed in young leaves (Yu et al. 2020). Young leaves that grow in high-light environments do not have sufficient ability to utilize more light energy, which will lead to the formation of excess light energy, which induces the production of ROS and causes the young leaves to be damaged by photoinhibition. Therefore, it is necessary for young leaves to accumulate a large amount of photoprotective substances to avoid such damage. This explains why %Anthocyanins, %Flavonoids, %Phenolics, %TAC and %Car/Chl were significantly negatively correlated with %Pn (Figures 4a–d and h, and 6b–d). Interestingly, the process of increasing photosynthetic capacity was not always parallel to the process of increasing chlorophyll content. The accumulation of about 50% of chlorophyll was also a key period to distinguish the changes of photosynthetic capacity. When the accumulation of chlorophyll reached ~50%, the photosynthetic capacity of leaves reached maximum levels. After that, the continuous accumulation of chlorophyll had little effect on the improvement of photosynthetic capacity (Figure 4e and f). The results of Chow et al. (2000) seem to confirm our results: when the chlorophyll content reached 50% of the final value, the light- and CO_2_-saturated Pmax and the light-limited quantum yield (QY) of O_2_ evolution (based on absorbed irradiance) in the absence of photorespiration reached their maximum values. In this sense, the results agree with our concept of 50% Chl defining the stage of photosynthetic maturity. We speculate that the reason is that plants' accumulation of more chlorophyll above the 50% level may be used to increase leaf absorptance, thereby increasing the efficiency of utilizing incident light for photosynthesis.

This study has some limitations. It did not measure photosynthesis under light-limited conditions, so it is not clear whether the light-limited photosynthetic efficiency continues to increase after 50% chlorophyll accumulation. Therefore, the definition of photosynthetic maturity needs to be further scrutinized in future work. This study only provides a preliminary prediction that the accumulation of chlorophyll at ~50% may be a sign of photosynthetic maturity in plants.

## Conclusions

**Figure 7:**
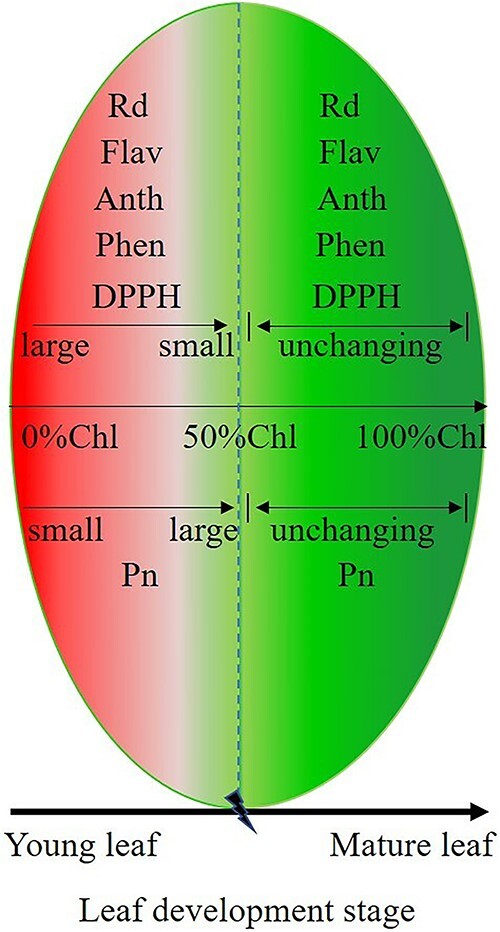
Schematic diagram of photoprotective capacity and photosynthetic capacity with photosynthetic pigment changes during leaf development in evergreen trees of subtropical forests.

Overall, this study evaluated the dynamic changes in the photoprotection ability and photosynthetic maturation of leaves at different developmental stages in subtropical forests by randomly collecting eight dominant tree species and using the spatio-temporal replacement method. The following three conclusions were reached:

(i) Anthocyanins, flavonoids and total phenolics played an important role in photoprotection during leaf development. However, anthocyanins, flavonoids and total phenolics have different photoprotection mechanisms. Anthocyanins mainly played the role of light attenuation, while flavonoids and total phenolics mainly played the role of antioxidation. Moreover, it appears that anthocyanins generally play a role in light attenuation in most plant leaves.
(ii) The process of leaf maturation was actually a process of transformation: the transformation from high photoprotective ability, high metabolic capacity and low photosynthetic capacity to low photoprotective capacity, low metabolic capacity and high photosynthetic capacity. With increasing leaf maturity, the photoprotective ability and Rd gradually decreased, and the photosynthetic ability gradually increased (Figure 7).
(iii) When the chlorophyll in plant leaves accumulated to ~50% of the final value, the anthocyanins disappeared almost completely, and the flavonoid content, total phenolic content, TAC and Rd decreased to the lowest level, while Pn reached the highest level (Figure 7). In summary, it was predicted in this study that the accumulation of ~50% chlorophyll may be a sign of plant photosynthetic maturation.

## Data Availability

The data that supports the findings of this study are available within the article and its Supporting Information.
